# Group 1 Allergen Genes in Two Species of House Dust Mites, *Dermatophagoides farinae* and *D. pteronyssinus* (Acari: Pyroglyphidae): Direct Sequencing, Characterization and Polymorphism

**DOI:** 10.1371/journal.pone.0114636

**Published:** 2014-12-10

**Authors:** Rubaba Hamid Shafique, Pavel B. Klimov, Muhammad Inam, Farhana Riaz Chaudhary, Barry M. OConnor

**Affiliations:** 1 Department of Zoology, Pir Mehr Ali Shah Arid Agriculture University Rawalpindi, Rawalpindi, Pakistan; 2 Department of Ecology and Evolutionary Biology, University of Michigan, Ann Arbor, Michigan, United States of America; 3 Faculty of Biology, Tyumen State University, Tyumen, Russia; 4 Physiology Laboratory, Department of Zoology, University of the Punjab, Lahore, Pakistan; Kermanshah University of Medical Sciences, Iran, Republic of Islamic

## Abstract

Group 1 allergens of *Dermatophagoides farinae* (Der f 1) and *D. pteronyssinus* (Der p 1) dominate overall allergic responses in house dust mite allergy patients. The need for accurate identification and characterization of representative variants of group 1 allergens in any given geographic locality has been emphasized for development of appropriate allergen extracts. Regional amino acid sequence polymorphism has been described but the extent of this polymorphism is not well understood. Such data are completely absent for the USA and many other countries. Most previous studies used cDNA libraries generated by reverse transcriptase (RT-PCR) and/or primers amplifying shorter fragments of this gene. Using novel species-specific primers and direct PCR, we document group 1 allergen gene sequence polymorphism in populations of *D. farinae* and *D. pteronyssinus* from the USA and Pakistan. We report two novel introns (nt pos 87 and 291) in both species, and the absence of intron 3 in Der p 1. Thirteen silent and one novel non-synonymous mutation (Tryptophan W^197^ to Arginine R^197^) were detected in *D. farinae*. The potential medical significance of the latter mutation is discussed. Two haplotypes of the Der f 1 gene were identified, haplotype 1 (63%) was more frequent than haplotype 2 (18%). Polymorphism in Der f 1 displayed geographical localization, since both haplotypes were present in mite populations from Pakistan whereas haplotype 1 was observed only in the USA. In Der p 1, a silent mutation at nt (aa) position 1011(149) and four non-synonymous mutations at positions 589(50), 935(124), 971(136), 1268(215) were observed. These mutations were reported from many other geographic regions, suggesting that polymorphism in the Der p 1 gene is panmictic. The extent of polymorphism in both genes is substantially lower than that reported previously (0.10–0.16% vs 0.31–0.49%), indicating the need for careful evaluation of potential polymerase errors in studies utilizing RT-PCR.

## Introduction

Pyroglyphid house dust mites of the genus *Dermatophagoides* are important sources of allergens in the indoor environment of human dwellings [1], causing allergic diseases such as asthma, rhinitis and atopic dermatitis, in millions of people worldwide [2]. Over 30 different proteins and macromolecules are known to produce IgE-binding reactions in patients allergic to house dust mites [3]. Among these molecules, group 1 allergens of *Dermatophagoides farinae* and *D. pteronyssinus* (Der f 1 and Der p 1) dominate overall allergic responses [3]–[5]. Group 1 allergens are cysteine proteases (proteolytic enzymes) [6]–[8], having the ability to induce pro-inflammatory response by breaking lung epithelium [9], [10]. Earlier reports described local variants of group 1 allergens from different geographical regions in Thailand, Korea, China, Australia, and the UK [11]–[20]. Variation data reports come from predicted amino acid sequences based on either amplification from genomic DNA [20], [21] or, more frequently, cDNA libraries obtained through reverse transcriptase polymerase chain reaction (RT-PCR) and subsequent cloning [11], [14], [16], [21]. Because reverse transcriptase is not proof reading [22], it is not surprising that a higher number of mutations were reported in the latter studies.

Amino acid sequence variations can influence IgE binding reactivity of allergens [23]. Single amino acid mutations can alter inflammatory cytokine production of T cells specific for Der p 1 [24], [25]. It is possible that these mutations influence the inherent allergenicity of particular variants and contribute to differential IgE binding frequency by increasing diversity of epitopes [26].

Der f 1 and Der p 1 share 81% amino acid sequence identity [27]–[29] (83%, our data), due to which cross reactivity exists among these two allergens [27]–[29]. Despite this relatively high degree of homology and cross reactivity, monoclonal antibodies (mAbs) produced against Der p 1 and Der f 1 are species specific [27], [28]. This contradictory behavior may be attributed to the position of IgE binding epitopes in allergen molecules. Epitope residues that are present in high homology regions of the allergen explain cross reactivity between Der f 1 and Der p 1 allergens, while the part of the IgE binding epitope found in variable regions may result in species specificity. An example of the first case is 4C1 anti Der f 1 mAb [28], which binds to a cross reactive (conserved) epitope on both Der f 1 and Der p 1. This set of amino acids includes Glutamic acid (E)^14^, Aspartic acid (D)^16^, Arginine (R)^18^, Serine (S)^19^, Arginine (R)^21^, Glycine (G)^156^, Arginine (R)^157^, Isoleucine (I)^159^, Threonine (T)^181^, Glutamine (Q)^182^, Tyrosine (Y)^186^, Aspartic acid (D)^199^, Tyrosine (Y)^202^ and Tyrosine (Y)^204^. The second part of this epitope is a calcium (Ca^+^) binding residue on the allergen molecule comprising four amino acids: Aspartic acid (D)^57^, Leucine (L)^58^, Glutamic acid (E)^60^ and Glutamic acid (E)^92^ [30]. Analysis of these amino acid residues may help to predict cross reactivity in allergens from different mite species. As mutations in some IgE binding epitopes may affect both cross-reactivity and specificity of monoclonal antibodies, allergen diversity both among and within species should be taken into consideration for development of appropriate allergen extracts. This accentuates the need for accurate identification and characterization of representative variants in any given geographic locality. Regional amino acid sequence polymorphism and the extent of this polymorphism are poorly studied, and such data are completely absent for many countries, including the USA and Pakistan. Our paper is a study of within- and among- species polymorphism in the group 1 allergen gene in two medically important species of house dust mites (*Dermatophagoides farinae* and *D. pteronyssinus*) collected in the USA and Pakistan. We compare results (direct PCR) with previous data (RT-PCR) and provide a comparative analysis of group 1 allergen peptide sequences of different mite species.

## Materials and Methods

### Collection of dust samples

Samples were obtained from domiciliary dust of beds and sofas in Pothwar, Pakistan (33.60°N 73.03°E) during July 2012. Sampling was done with a household vacuum cleaner specially modified to collect dust according to a previously described protocol [31]. Mites were isolated using the saturated sodium chloride floatation method [32]. The supernatant was filtered through 45µm filter paper. Mites were removed with a mounting needle under a dissecting microscope and stored in 70% ethanol at −20°C (later transferred into 96% ethanol). Specimens of *D. farinae* from the USA were obtained from a laboratory culture maintained at the University of Michigan, Museum of Zoology (started from multiple specimens collected locally 42.27°N 83.73°W in 2005). Specimens of *D. pteronyssinus* originated from cultures in Greer Laboratories, North Carolina, USA. Exact collection localities are available as GenBank metadata deposited along with our sequences.

### DNA extraction, amplification, sequencing, and alignment

DNA was extracted with a QIAamp DNA Micro Kit (Qiagen). A single mite specimen was isolated with a mounting needle and placed on a drop of lysis buffer (Buffer ATL) in a cavity glass slide. Under a dissection microscope, the specimen was teased to break the exoskeleton and then transferred into a 1.5 ml microcentrifuge tube containing 180 µl of buffer ATL. The remaining steps followed the manufacturers’ protocol for tissue samples. DNA was eluted in 30µl of AE buffer and stored at −20°C.

Der p 1 and Der f 1 genes were amplified by nested PCR. For each species, two sets of species-specific primers were designed in Primer3 to amplify almost the entire coding region of the gene [33]. For uniform sequencing, T3 and SP6 tails were added to the forward inner and reverse inner primers respectively (Table 1). PCR was performed in 20 µl volume with Platinum Taq DNA Polymerase (Invitrogen). The master mix for initial PCR contained 2.0 µl of PCR buffer, 1.4 µl MgSO_4_ (50 mM) and dNTPs (10 mM each), 0.8 µl of primers (10 µM of each forward and reverse), 0.12 µl of Platinum Taq polymerase (1.5U) and 0.6–1 µl of DNA template (depending on DNA concentration in the sample), the total volume was made up to 20 µl with distilled water. The thermocycler protocol was as follows: 94°C, 2 min; [94°C, 30 sec; 48°C, 35 sec; 72°C, 2 min]×35 cycles; 72°C for 7 min. PCR products were kept at 4°C until the second PCR was performed. For the second PCR (inner primers) the master mix was modified with a reduced quantity of Taq Polymerase 0.08 µl (1.0 U) and 0.6 µl of PCR products from the first PCR. The thermocycler protocol was set as above, except for the annealing step (50°C for 38 sec), the extension step (1.50 min), and the total number of cycles (38). PCR products were run on 1.5% agarose gel, bands were excised under UV light, and DNA was purified with a QIAquick gel extraction kit (Qiagen).

**Table 1:** Species-specific oligonucleotide primers used in nested amplification of the group 1 allergen gene (Der f 1 and Der p 1).

Primer Name	Primer Sequence (5′ to 3′)
DF_Der1_P_121F	AAAATTCATCAAAAATGAAATTCG
DF_Der1_P_1436R	CTCGCAAGAGTAGTTGTTTTTATTTTG
DP_Der1_P_108F	CTCTCTAAAATCTAAAATCCATCC
DP_Der1_P_1509R	AATTTAATTTTTGTGAATG
DF_Der1_P.Ch_133F_T3	ATTAACCCTCACTAAAGGGA ATGAAATTCGTTTTGGCCATTG
DF_Der1_P.Ch_1430R_SP6	ATTTAGGTGACACTATAG CGCAAGAGTAGTTGTTTTTATTTTGA
DP_Der1_P_Ch.115F_T3	ATTAACCCTCACTAAAGGGA AAAATCTAAAATCCATCCAACATGA
DP_Der1_P_Ch.1458R_SP6	ATTTAGGTGACACTATAG TTTTAAATAAATTAGTGACAATCA

In primer names: DF - *Dermatophagoides farinae*, DP - *D. pteronyssinus*; P – outer primer; P.Ch. – inner primer; F – forward; R – reverse; T3– has T3 tail (underlined in primer sequence); SP6– has SP6 tail (underlined).

Sequencing was done with an Applied Biosystems 3730 XL DNA Sequencer at the University of Michigan sequencing core. Sequences were analyzed in Sequencher ver. 5.0. Because our primers overlap small coding fragments at the 5′ end of the gene, our sequences were partial (22 nt missing for Der f 1 and 4 nt missing for Der p 1 at the 5′ end). These sequences were submitted to GenBank, accession numbers KJ542064 through KJ542097 (Table 2). However, in this paper, for simplicity, we assume that our sequences are complete and use GenBank data for cDNA of preproenzyme (AB034946 for Der f 1 and U11695.1for Der p 1) for the short missing 5′ ends. Thus, position 1 of our alignment coordinates is the first nucleotide of the start codon. Intron identification and translation into polypeptide was done in Mesquite ver. 2.75. After computer-assisted translation, preproenzyme sequences (full length peptides) were obtained. Three regions on the peptides were identified: signal or leader peptide (pre), inactive enzyme (proenzyme) and mature enzyme. In amino acid sequences, the starting position was set to the 1^st^ amino acid of the mature peptide, whereas the signal peptides and proenzyme regions were given negative coordinates [34]. Homologous group 1 allergen DNA and protein sequences were retrieved from the National Centre for Biotechnology Information (NCBI) nucleotide, EST, and protein databases using blastn and blastp. Resulting sequences, *Euroglyphus maynei* (AAC82351), *Psoroptes ovis* (CAK32515), *Sarcoptes scabiei (*AAS93667), *Acarus siro (*ABU50820), *Blomia tropicalis* (AAQ24541) and *Tyrophagus putrescentiae (*ABM53753), were aligned with *D. farinae* (KJ542065) and *D. pteronyssinus* (KJ542087). Alignment was done in Clustal Omega. Signal peptide prediction of all selected cysteine proteases was done in SignalP ver4.1 [35]. The 3-dimensional structure and function prediction of selected proteins was carried out by iterative threading assembly refinement (I-TASSER) server [36]–[38].

**Table 2:** Exons, introns, and sequence polymorphism in the group 1 allergen-encoding gene of two species of house dust mites from Pakistan and the USA.

Gene Map		*Exon 1*	*Intron 1*		*Exon 2*	*Intron 2*	*Exon 3*		*Intron 3*			*Exon 4*	*Intron 4*				*Exon 5*						*Intron 5*	*Exon 6*		
Mutation Sites			*a*	*b*		*c*	*d*	*e*	*f*	*g*	*h*	*i*	*J*	*k*	*l*	*m*	*n*	*o*	*p*	*q*	*r*	*s*	*t*	*u*	*v*	*w*
Alignment Coordinates, nt			113	152		312	589	600	680	686	718	794	824	829	842	843	935	971	978	996	1011	1014	1052	1207	1211	1268
Protein coordinates Df(Dp)							5*1(50)*	54(55)				100(99)					125(124)	137(136)	139(138)	145(144)	150(149)	151(150)		195(194)	197(196)	216(215)
Translated Amino Acids (triplet codes)							Tyr(TAC)/His(CAC)	Thr (ACG/ACT)				Arg (CGA/AGA)					Ala(GCA)/Val(GTA)	Ser(AGC)/Thr(ACC)	Ile (ATT/ATC)	Ile (ATT/ATC)	Ala (GCA/GCT)	Phe (TTT/TTC)		Thr (ACT/ACA)	Trp(TGG)/Arg(CGG)	Glu(GAA)/Gln(CAA)
***D. farinae***																										
**RS17 (KJ542065)**	**PK**	**No Mutations**	**-**	A	**No Mutations**	**-**	**-**	ACG	G	T	C	CGA	**-**	**-**	C	G	**-**	-	**AT** T	ATT	**-**	TTT	T	ACT	TGG	-
**RS20 (KJ542066)**	**PK**		**-**	A		**-**	**-**	ACG	G	T	C	CGA	**-**	**-**	T	G	**-**	-	**AT** T	ATT	**-**	TTT	T	ACT	TGG	-
**RS25 (KJ542067)**	**PK**		**-**	A		**-**	**-**	ACG	G	T	C	CGA	**-**	**-**	T	G	**-**	-	**AT** T	ATT	**-**	TTT	T	ACT	TGG	-
**RS26 (KJ542068)**	**PK**		**-**	W		**-**	**-**	ACK	G	Y	M	CGA	**-**	**-**	C	G	**-**	-	**AT** T	ATY	**-**	TTY	T	ACW	TGG	-
**RS27 (KJ542069)**	**PK**		**-**	T		**-**	**-**	ACT	G	C	A	CGA	**-**	**-**	C	T	**-**	-	**AT** T	ATC	**-**	TTC	T	ACA	TGG	-
**RS29 (KJ542070)**	**PK**		**-**	A		**-**	**-**	ACG	G	T	C	CGA	**-**	**-**	T	G	**-**	-	**AT** T	ATT	**-**	TTT	T	ACT	TGG	-
**RS30 (KJ542071)**	**PK**		**-**	T		**-**	**-**	ACT	G	C	A	CGA	**-**	**-**	C	T	**-**	-	**AT** T	ATC	**-**	TTC	T	ACA	TGG	-
**RS31 (KJ542072)**	**PK**		**-**	T		**-**	**-**	ACT	G	C	A	CGA	**-**	**-**	C	T	**-**	-	ATC 2	ATC	**-**	TTC	T	ACA	CGG^3^	-
**RS33 (KJ542073)**	**PK**		**-**	A		**-**	**-**	ACG	G	T	C	CGA	**-**	**-**	T	G	**-**	-	**AT** T	ATT	**-**	TTT	T	ACT	TGG	-
**RS34 (KJ542074)**	**PK**		**-**	A		**-**	**-**	ACG	G	T	C	CGA	**-**	**-**	T	G	**-**	-	**AT** T	ATT	**-**	TTT	T	ACT	TGG	-
**RS35 (KJ542075)**	**PK**		**-**	T		**-**	**-**	ACT	G	C	A	CGA	**-**	**-**	C	T	**-**	-	**AT** T	ATC	**-**	TTC	T	ACA	TGG	-
**RS36 (KJ542076)**	**PK**		**-**	T		**-**	**-**	ACT	G	C	A	CGA	**-**	**-**	C	T	**-**	-	**AT** T	ATC	**-**	TTC	T	ACA	TGG	-
**RS37 (KJ542077)**	**PK**		**-**	A		**-**	**-**	ACG	G	T	C	CGA	**-**	**-**	T	G	**-**	-	**AT** T	ATT	**-**	TTT	T	ACT	TGG	-
**RS40 (KJ542078)**	**PK**		**-**	T		**-**	**-**	ACK	G	C	M	CGA	**-**	**-**	C	K	**-**	-	**AT** T	ATY	**-**	TTY	T	ACW	TGG	-
**RS41 (KJ542079)**	**PK**		**-**	A		**-**	**-**	ACG	G	T	C	AGA^2^	**-**	**-**	T	G	**-**	-	**AT** T	ATT	**-**	TTT	T	ACT	TGG	-
**RS42 (KJ542080)**	**PK**		**-**	A		**-**	**-**	ACG	R	T	C	CGA	**-**	**-**	T	G	**-**	-	**AT** T	ATT	**-**	TTC	A	ACT	TGG	-
**RS03 (KJ542064)**	**USA**		**-**	A		**-**	**-**	ACG	G	T	C	CGA	**-**	**-**	T	G	**-**	-	**AT** T	ATT	**-**	TTT	T	ACT	TGG	-
**RS59(KJ542081)**	**USA**		**-**	A		**-**	**-**	ACG	G	T	C	CGA	**-**	**-**	T	G	**-**	-	**AT** T	ATT	**-**	TTT	T	ACT	TGG	-
**RS60(KJ542082)**	**USA**		**-**	A		**-**	**-**	ACG	G	T	C	CGA	**-**	**-**	T	G	**-**	-	**AT** T	ATT	**-**	TTT	T	ACT	TGG	-
**RS61(KJ542083)**	**USA**		**-**	A		**-**	**-**	ACG	G	T	C	CGA	**-**	**-**	T	G	**-**	-	**AT** T	ATT	**-**	TTT	T	ACT	TGG	-
**RS62(KJ542084)**	**USA**		**-**	A		**-**	**-**	ACG	G	T	C	CGA	**-**	**-**	T	G	**-**	-	**AT** T	ATT	**-**	TTT	T	ACT	TGG	-
**RS63(KJ542085)**	**USA**		**-**	A		**-**	**-**	ACG	G	T	C	CGA	**-**	**-**	T	G	**-**	-	**AT** T	ATT	**-**	TTT	T	ACT	TGG	-
***D.pteronyssinus***																										
**RS12 (KJ542087)**	**PK**		**T**	-		**G**	TAC	-	**Intron 3 absent**			**-**	**C**	**A**	-	-	**G** CA	AGC	**-**	-	**GC** A	-	-	-	**-**	GAA
**RS16 (KJ542088)**	**PK**		**T**	-		**G**	TAC	-				**-**	**C**	**A**	-	-	**G** TA	AGC	**-**	-	**GC** A	-	-	-	**-**	GAA
**RS18 (KJ542089)**	**PK**		**T**	-		**G**	TAC	-				**-**	**C**	**A**	-	-	**G** TA	AGC	**-**	-	**GC** A	-	-	-	**-**	GAA
**RS19 (KJ542090)**	**PK**		**C**	-		**R**	TAC	-				**-**	**C**	**R**	-	-	**G** CA	AGC	**-**	-	**GC** W	-	-	-	**-**	GAA
**RS24 (KJ542091)**	**PK**		**T**	-		**G**	TAC	-				**-**	**C**	**A**	-	-	**G** CA	AGC	**-**	-	**GC** A	-	-	-	**-**	GAA
**RS65 (KJ542095)**	**PK**		**T**	-		**G**	TAC	-				**-**	**C**	**A**	-	-	**GTA**	AGC	**-**	-	**GC** A	-	-	-	**-**	GAA
**RS68 (KJ542096)**	**PK**		**T**	-		**G**	TAC	-				**-**	**C**	**A**	-	-	**G** YA	AGC	**-**	-	**GC** A	-	-	-	**-**	GAA
**RS73 (KJ542097)**	**PK**		**T**	-		**G**	TAC	-				**-**	**C**	**A**	-	-	**G** YA	AGC	**-**	-	**GC** A	-	-	-	**-**	GAA
**RS6 (KJ542086)**	**USA**		**T**	-		**G**	TAC	-				**-**	**C**	**R**	-	-	**G** CA	AGC	**-**	-	**GC** A	-	-	-	**-**	GAA
**RS45 (KJ542092)**	**USA**		**T**	-		**G**	TAC	-				**-**	**C**	**A**	-	-	**G** CA	AGC	**-**	-	**GC** A	-	-	-	**-**	GAA
**RS51(KJ542093)**	**USA**		**T**	-		**G**	CAC	-				**-**	**T**	**A**	-	-	**G** CA	ACC	**-**	-	**GC** T	-	-	-	**-**	CAA
**RS52 (KJ542094)**	**USA**		**T**	-		**G**	TAC	-				**-**	**C**	**A**	-	-	**G** CA	AGC	**-**	-	**GC** A	-	-	-	**-**	GAA
**GenBank data**			**Intron** 1			**Intron** 1	**TAC/CAC**	**ACT/ACG**	**G**	**C**	**A**	**CGA**	**C**	**A**	**C**	**T**	**GCA/GTA**	**AGC/ACC**	**ATT/ATG**	**ATT/ATC**	**GCT/GCA**	**TTT/TTC**	**T**	**ACT**	**TGG**	**GAA/CAA**
**Novel Mutations**			**T/C**	**A/T**		**G/A**			**A**	**T**	**C**	**AGA** 2	**T**	**G**	**T**	**G**			**ATC** 2				**A**		**CGG** 3	

PK: Pakistan; USA: United States of America;

1 not reported in previous studies;

2 Novel silent (synonymous) mutation;

3 Novel non silent (non-synonymous) mutation. GenBank accession numbers for the sequences resulted from our study are given in parentheses.

## Results

### Der f 1 gene polymorphism

**Figure 1:**
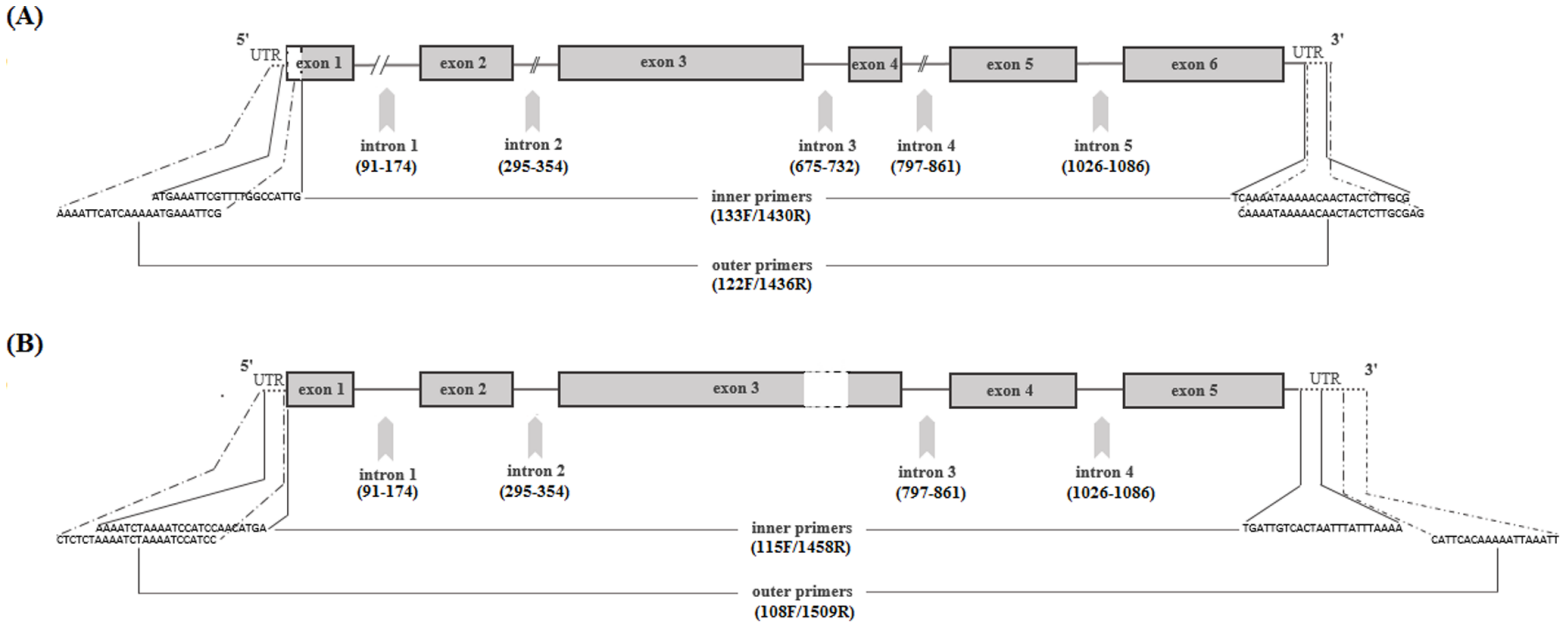
Gene map of Der f 1 (A) and Der p 1 (B) allergens amplified by nested PCR. UTR: untranslated regions; (numbers): nucleotide position for introns. The blank region with dashed outlines at exon 1 indicates partial 5′end of genes (given our sequences). T3/SP4 tails (see table 1) in primer sequences are not shown.

Twenty-two Der f 1 gene sequences, including six from the USA, were analyzed. The length of the gene (from start to stop codon), including six exons and five introns was 1278 base pairs (bp). Of the five introns we detected, two (intron1 and 2, at mRNA nucleotide positions (nt. pos) 87 and 291, respectively) were not reported previously (Table 2, Fig. 1). Seven mutations were observed in non-translated regions (introns) of the gene (Table 2).

**Figure 2:**
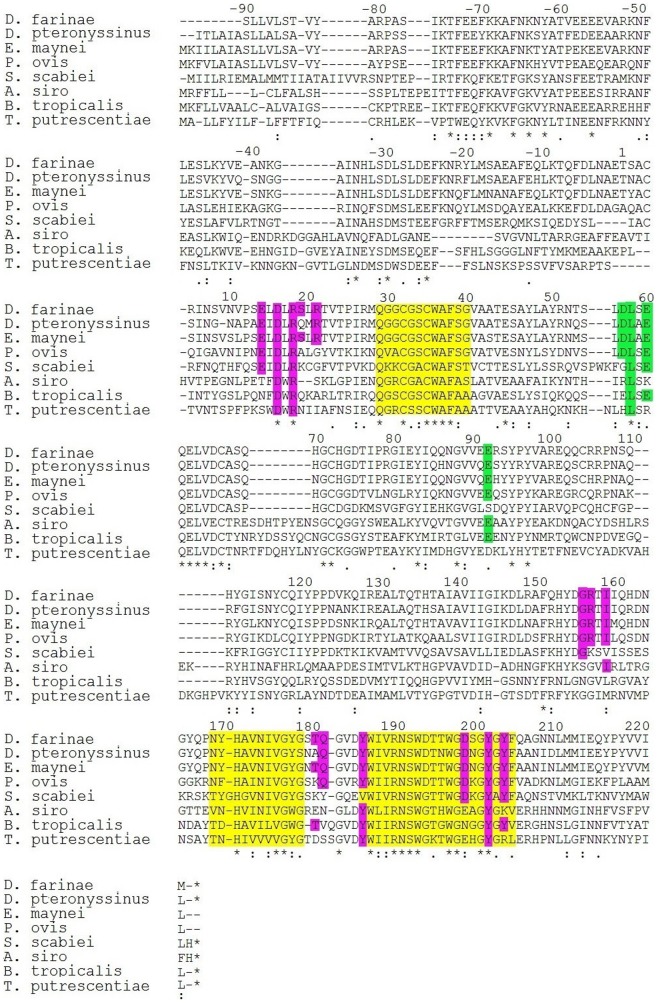
Alignment of group 1 allergens (Cysteine proteases) of selected mite species. Active site regions are shown in yellow, amino acid residues making 4C1 binding epitope (pink) and calcium binding epitope (green); “*”: identical, “:”: conserved and “.”: semi-conserved sites.

**Figure 3:**
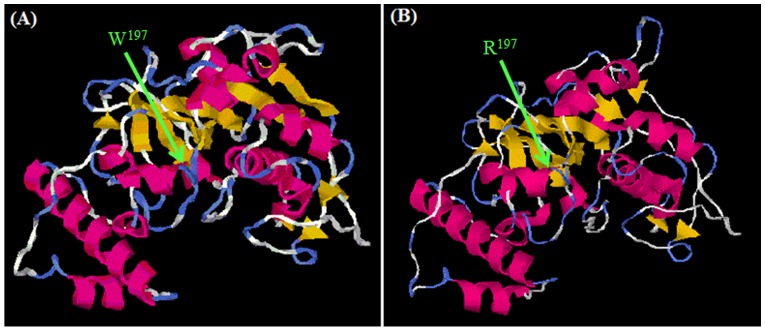
I-TASSER result showing tertiary structure predictions for RS33 (Tryptophan variant) and RS31 (Arginine variant) of Der f 1 protein [36]–[38]. A: specimen RS33; B: specimen RS31.

All *D. farinae* sequences show 99–100% homology, with two distinct haplotypes (H1, H2, S1 Figure). Haplotype 1 (e.g., RS17 and RS20) was the more frequent (63.5%, 14/22), followed by haplotype 2 (18%, 4/22, e.g., RS27 and RS30) and heterozygous variants (18%, 4/22, e. g., RS26 and RS40). Der f 1 sequences of all USA specimens (e.g., RS03) were identical to the H1 sequence from the Pakistan population (S1 Figure). Mutations were observed at 14 different positions along the whole length of the sequenced gene, where seven mutations were in the introns (non-translated region). Mutations (substitutions) in the exons were at nt pos 600, 794, 978, 9926, 1014, 1207 and 1211. Table 2 shows the corresponding amino acid positions and translated amino acid at each substitution site. Of these, all but one mutation were silent. The single non-silent mutation was observed in variant RS31 (KJ542072) where amino acid Tryptophan (W) was substituted by Arginine (R) at nt(aa) pos 1211(197) (Table 2). This novel mutation occurred in the active site of the mature enzyme (Fig. 2). Secondary and tertiary protein structure prediction indicated slight difference between these two variants whereas no predicted function difference was observed (Fig. 3).

### Der p 1 gene polymorphism

Twelve Der p 1 gene sequences were obtained, including four from the USA. The length of the gene (from start to stop codon), including five exons and four introns was 1248–1250 bp, owing to a variable poly-T region in intron1 (S2 Figure). The key difference between Der p 1 and Der f 1 genes is the absence of intron 3 (length 58 nt) in the Der p 1 gene (Fig. 1). Although intron 3 was known for Der f 1, no such data existed for Der p 1. Four mutations were observed in non-translated regions (introns) (Table 2). *D. pteronyssinus* samples obtained from USA and Pakistan displayed polymorphism at five nucleotide positions in the coding regions (exons). Deduced amino acid sequences showed four non-synonymous substitutions at positions [nt (aa)]: 589 (50), 935(124), 971(136), 1268(215) and a synonymous mutation at 1011(149) (Table 2). In contrast to *D. farinae*, all the sequences of Der p 1 were unique, differing by 1–2 amino acid residues, but no distinct haplotypes were observed (S2 Figure). Comparison of our results and data from previous studies showed that mutations in Der p 1 aa pos (Y->H)^50^, (V->A)^124^, (T->S)^136^ and (Q->E/K/G)^215^ were most frequently reported (Table 3). Nearly 20 other mutations that were sporadically observed in Der p 1 previously [11]–[20] were not found in our survey.

**Table 3:** Geographical polymorphism in the Der p 1 allergen.

Locality and DNA template	Most frequently reported mutation sites				Other sporadic mutation sites	Reference
	Y^50^	V^124^	T^136^	Q^215^		
Pakistan genomic	-	V/A	S	E	None	This study
USA genomic	Y/H	A	-	-	None	This study
UK genomic	-	A	S	n.r.	None	Kent et al., 1992
Sydney genomic	-	V/A	S/T	E	None	Smith et al., 2001
Melbourne cDNA	H/Y	V/A	S/T	E/Q	1	Chua et al., 1993
Perth cDNA	-	V/A	S	E/K	6	Smith et al., 2001
Bangkok cDNA	H/Y	A/V	S	E/G	9	Piboonpocanun et al., 2006
Korea cDNA	Y/H	V/A	S	E	20	Jeong et al., 2012

Superscript on amino acid symbol indicates the position in polypeptide (N.R.: not reported; “a.a/a.a”: higher frequency/lower frequency reported; “-”: no mutation

### Polypeptide analysis

**Figure 4:**
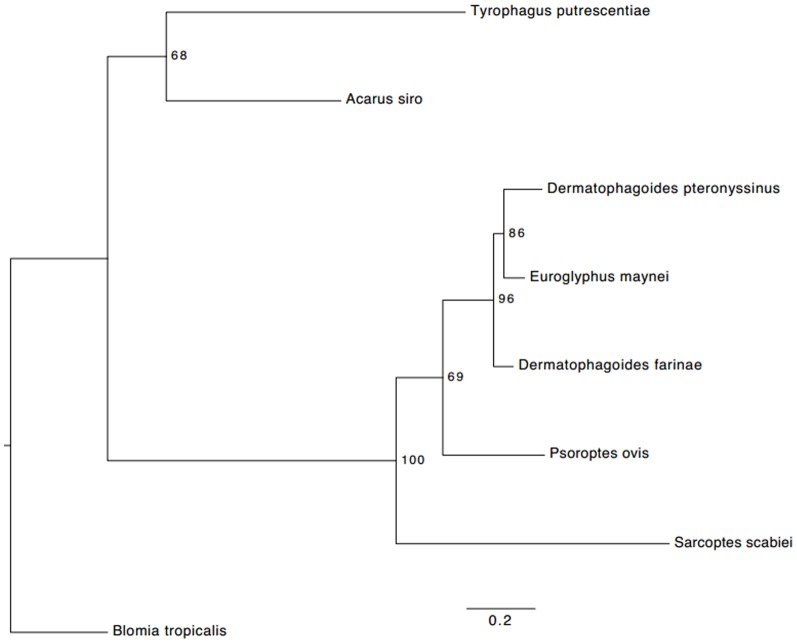
Maximum likelihood tree of the group 1 allergen protein of acariform mites. For each node, nonparametric bootstrap support values (100 replicates) are indicated. The tree was inferred in RAxML ver.7.5.5 [39] using the WAGF model of amino acid evolution as found in PartitionFinder ver. 1.1.1 [40].

The percent identity tree of group 1 allergens shows close similarity of pyroglyphid dust mites (*D. farinae, E. maynei* and *D. pteronyssinus*) with the psoroptid mange mite, *P. ovis* (Psoroptidae); whereas large phylogenetic distances were found between the pyroglyphid mites and species from the storage mite families Echimyopodidae (*B. tropicalis*) and Acaridae (*A. siro* and *T. putrescentiae*) (Fig. 4, Table 4). A closer evolutionary relationship of *E. maynei* with *D. pteronyssinus* was observed in the tree whereas *D. farinae* and *D. pteronyssinus* were more distantly placed (Fig. 4).

**Table 4:** Percent identity matrix of aligned group1 allergens.

		Der f 1	Der p 1	Eur m 1	Pso o 1	Sar s 1	Aca s 1	Blo t 1	Tyr p 1
1.	Der f 1	100.00	-	-	-	-	-	-	-
2.	Der p 1	83.01	100.00	-	-	-	-	-	-
3.	Eur m 1	86.58	84.59	100.00	-	-	-	-	-
4.	Pso o 1	64.86	62.58	64.49	100.00	-	-	-	-
5.	Sar s 1	44.98	44.90	44.30	43.85	100.00	-	-	-
6.	Aca s 1	35.67	33.00	35.29	35.95	27.30	100.00	-	-
7.	Blo t 1	35.41	34.19	37.38	33.87	30.97	42.37	100.00	-
8.	Tyr p 1	32.01	29.97	30.65	30.00	27.42	39.38	38.23	100.00

Der f 1: D. farinae; Der p 1: D. pteronyssinus; Eur m 1: E. maynei; Pso o 1: P. ovis; Sar s 1: S. scabiei; Aca s 1: A. siro; Blo t 1: B. tropicalis; Tyr p 1: T. putrescentiae.

**Figure 5:**
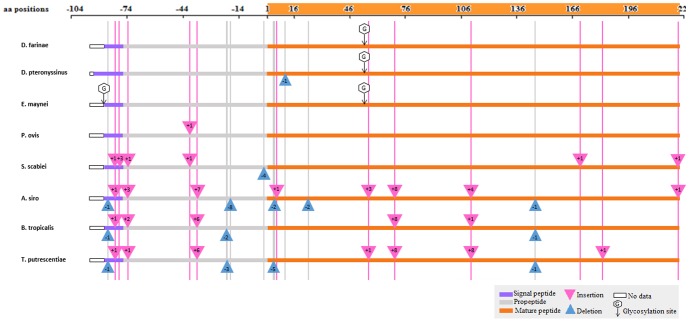
Translated Der f 1 polypeptide aligned with cysteine proteases of other mite species: *Dermatophagoides pteronyssinus* (Der p 1: present study, and U11695.1for Der p 1 to complete our partial signal peptide sequence), *Euroglyphus maynei* (Eur m 1:AAC82352.), *Psoroptes ovis* (Pso o 1:Q1EIQ3.1), *Sarcoptes scabiei* (Sar s 1: AAS93667.1), *Acarus siro* (Acr s: ABU50820.1), *Blomia tropicalis* (Blo t 1: AAQ24541.1) and *Tyrophagus putrescentiae* (Tyr p 1: ABM53753.1). Numbers inside triangles indicate the number of amino acids (aa). The “No data” box indicates missing 5′ ends.). AB034946 sequence was used to complete our partial Der f 1 signal peptide sequence.

The total length of the translated Der f 1 polypeptide was 321 amino acids. This included the signal peptide (with the C-terminus at pos −81), the proenzyme part (80 amino acids, pos −80 to −1), and the mature enzyme (223 amino acids, pos 1 to 223). Coordinates for Der p 1 were similar, except for the mature enzyme, which had a single deletion at aa pos 9, therefore, its length was 222 amino acids (Fig. 5).

The cleavage sites for signal peptides predicted for all cysteine proteases in this study were between aa pos −80 and −81. The length of signal peptides in Der f 1, Der p 1, Eur m 1, Pso o 1 and Blo t 1 was 18 amino acids, whereas Sar s 1, Aca s 1 and Try p 1 were 24, 15 and 17 amino acids long, respectively. Identification of proenzyme regions was based on the length of the signal peptide and mature enzyme (Fig. 5). No insertions or deletions were found in the Eur m 1 protein, whereas in Pso o 1 there was an insertion between aa pos −40 and −41 in the proenzyme region. In Sar s 1, Aca s 1, Blo t 1 and Tyr p 1, 6 to 7 insertions and 1 to 5 deletions were observed (Fig. 5).

Forty-four identical amino acid residues were observed in the alignment. The active sites^13^ were well conserved: the first region (pos 29–40, length 11 aa) has an 80% mean similarity; the second (pos 170–180, length 11) 72%, and the third (pos 186–205, length 20) 79% mean identity (Fig. 2).

Amino acid residues making the 4C1 mAb binding epitope for Der f 1 were aligned with cysteine proteases of other mite species (Fig. 2, Table 5). Our analysis revealed its high identity with Eur m 1 (100%), Der p 1 (85.7%) and Pso o 1 (78.87%), varying from 2 to 3 amino acid residues, whereas the Ca^+^ ion binding region demonstrated a 100% identity (Fig. 2, Table 5).

**Table 5:** Comparison of group 1 allergen conserved amino acid residues involved in 4C1mAb and Ca^+^ binding epitopes in eight mite species.

	4C1 binding epitope														Ca^+^ Binding					
Amino acid Position	E^14^	D^16^	R^18^	S^19^	R^21^	G^156^	R^157^	I^159^	T^181^	Q^182^	Y^186^	D^199^	Y^202^	Y^204^	Identity score (%)	D^57^	L^58^	E^60^	E^92^	Identity score (%)
**Der f 1(ref)**	✓	✓	✓	✓	✓	✓	✓	✓	✓	✓	✓	✓	✓	✓	100	✓	✓	✓	✓	100
**Der p 1**	✓	✓	✓	Q	✓	✓	✓	✓	A	✓	✓	✓	✓	✓	85.71	✓	✓	✓	✓	100
**Eur m 1**	✓	✓	✓	✓	✓	✓	✓	✓	✓	✓	✓	✓	✓	✓	100	✓	✓	✓	✓	100
**Pso o 1**	✓	✓	✓	A	G	✓	✓	✓	K	✓	✓	✓	✓	✓	78.57	✓	✓	✓	✓	100
**Sar s 1**	✓	✓	✓	K	G	✓	K	V	K	Y	V	✓	✓	✓	50	G	✓	✓	S	50
**Aca s 1**	T	✓	✓	-	S	✓	G	✓	E	N	✓	E	✓	K	50	R	✓	K	✓	50
**Blo t 1**	N	✓	✓	Q	A	S	G	L	✓	V	✓	N	✓	✓	42.86	E	✓	✓	✓	75
**Tyr p 1**	S	✓	✓	N	I	N	G	M	D	S	✓	E	✓	R	28.57	H	✓	R	D	25

A high percent identity score (>50) was observed in Der p 1, Eur m 1, Pso o 1 compared to Der f 1, indicating possibility of cross-reactivity among these allergens.

## Discussion

Group 1 allergens of house dust mites are medically important since they show high IgE binding frequencies and are commonly used in diagnostic tests (e.g., the skin prick test) and immunotherapeutic management of house dust mite allergy patients. Polymorphism in group 1 allergens in different geographical regions has been of great concern because it may affect the efficacy of allergy tests and treatment of the allergic disease. GenBank data available to date are mostly based on cDNA libraries produced by amplifying mRNA using RT-PCR with subsequent cloning of PCR products. Unfortunately, these studies made little effort to distinguish between potential polymerase errors (a reverse transcriptase is non-proofreading) and actual sequence polymorphism. In this study, we employed direct gene amplification and sequencing of the two most important house dust mite species with the aim to reduce artifacts that may be introduced by the non-proofreading reverse transcriptase. This technique is also less labor intensive, so results can be obtained faster in future studies. Der f 1 allergen polymorphism observed in the present study shows two haplotypes. Haplotype 1 from the USA and Pakistan exactly matches with partial mRNA variants from Korea^12^ China^13^ and the UK^18^. In contrast, haplotype 2 detected in Pakistan specimens showed 100% similarity with variants reported from Thailand^9^ and China [15].

Almost all mutations (13) observed in our study were silent substitutions, however, there was one novel non-silent mutation (tryptophan to arginine) at aa pos 197. This mutation lies within the active region of the mature protein (Fig. 4) [39], [40]. Tryptophan (W) is an aromatic amino acid with a large side chain pointing into the core between α helices of the polypeptide. Its side chain makes many hydrophobic interactions. The amino acid arginine (R) is polar positively charged and can only make a few of these interactions, thus, potentially destabilizing the active site domain [41]. Although no significant change in structure and function was predicted, there still is a need to investigate the effect of this mutation on the properties of this peptide. This mutation might alter the enzyme activity of cysteine protease but since it does not lie in the IgE binding epitope residue, therefore, it may not affect the allergenic properties, immune response, and cross-reactivity of the protein. Further investigations may help to confirm this hypothesis.

In Der p 1, sporadic substitutions of amino acids have been reported previously [11]–[20]. However, at least some of them may actually represent artifacts introduced by polymerase errors. For example, only single occurrence of an amino acid substitution was reported at several aa positions: 19 (L->M), 21(P->T), 44 (D->E), 125(S->N), 129 (K->E) and 138 (M->I) [11], [21]. Immune response to polymorphic peptides with these substitutions was either reduced or absent, whereas polymorphic peptides with more frequent substitutions at aa pos 50 (Y->H), 124 (V->A), 136 (T->S) and 215(Q->E/K/G) were able to induce a T cell response, indicating their role in differential inflammatory cytokine production of T cells [21]. Additional, albeit indirect evidence for the presence of potential RT-PCR artifacts in published sequences is the substantial difference in percentages of mutations per sequenced nucleotide for GenBank cDNA data versus our data: 0.3071 vs 0.1614 for *D. farinae* and 0.4866 vs 0.0948 for *D. pteronyssinus*. These two lines of evidence support our argument that some clones reported in the literature may be artifacts of RT-PCR.

Chua et al (1993) reported six variants of Der p 1 from Australia using RT-PCR, including five non-synonymous (aa pos 50, 81, 124, 136 and 215) and one synonymous mutation aa pos 149 [16]. Results of our study coincide with five of these reported substitutions [16]. This probably indicates the panmictic nature of *D. pteronyssinus* populations. Mutations at aa pos 50(Y->H) and 124 (V->A) are the most frequent substitutions and have been shown to strongly affect the T cell response in humans and mouse [14], [16], [21]. It is now recognized that amino acids Y^50^, V^124^, T^136^ and Q^215^ are common in Der p 1 and Der f 1 at these sites. The effect of these amino acid substitutions needs to be studied in the future for the development of species-specific monoclonal antibodies.

The predicted Der f 1 allergen sequence in our study shows a high percent homology with Eur m 1 (85.58%) suggesting a closer phylogenetic relationship to Der f 1, although *D. farinae* and *D. pteronyssinus* are currently taxonomically classified in the same genus. However, recent molecular phylogenetic studies based on different genes also support the close relationship of *D. farinae* and *E. maynei* [42]. The ordered distances of group 1 allergen protein agree with the phylogenetic distances of these taxa inferred using five independent genes [42].

Similarly, there was 100% homology in the second active site residue (aa pos 186–205), IgE-binding epitopes and in the Ca^+^ binding epitopes of Der f 1 and Eur m 1. This is supported by earlier reports of a greater homology between these two mite allergens and evidence of cross reactivity between them [30], [43]. Der p 1 epitopes were also highly conserved (86%) where only serine (S^19^) was replaced by glutamine (Q). This may be the cause of cross reactivity reported earlier between Der f 1, Der p 1 and Eur m 1 [44]. Pso o 1 allergen also shows a 100% conservation of the Ca^+^ binding epitope residue, 79% 4C1 mAb epitope homology and 69% complete protein identity score. This explains the cross-antigenicity between allergens of house dust mites and other parasitic psoroptidians [45]. On the other hand, the complete absence of any cross reactivity between Der p 1 and Blo t 1 [46] is supported by the large phylogenetic distance between group 1 allergens of pyroglyphid and echimyopodid mites (*Blomia*).

In conclusion, our results indicate that very little polymorphism occurs in the group 1 allergen gene of *D. farinae*, where all but one mutation were silent and do not affect the primary structure of this protein. The discovery of a novel Trp→Arg mutation in the active site of the enzyme is the most exciting finding of our work. Further experiments are required to estimate the frequency of this novel Der f 1 allele. In this study, a substantial amino acid variation is present in *D. pteronyssinus*, but the number of variants is far fewer than reported earlier. In order to eliminate RT-PCR artifacts as a probable cause of these variations, we suggest that direct sequencing technique should be utilized in the study of genetic polymorphism. Polymorphism in Der f 1 gene did show some geographic distribution patterns; haplotype 1 is more common and widely distributed as compared to haplotype 2. Der p 1 gene polymorphism is panmictic and does not show any geographically localized variants. Our analysis of group 1 allergen proteins from different mite species confirms a close evolutionary relationship between pyroglyphids and parasitic psoroptid mange mites.

## Supporting Information

S1 Figure **Selected sequences of the Der f 1 gene showing to two distinct haplotypes:** Haplotype1-rows 1, 2 and 3 (RS20, RS17, RS03_USA); haplotype2-rows 4 and 5(Df_RS27 and Df_RS30); heterozygous - rows 6 and 7 (RS26 and RS40); and gb|Der-f1 gene (GenBank Accession number X65196).
(TIF)

S2 Figure **Selected Der p 1 gene sequences aligned in NCBI blastn. gb|Der-p1gene (GenBank accession number X65197.1).**
(TIF)
