# Supplementary figures and images for: Group 1 Allergen Genes in Two Species of House Dust Mites, Dermatophagoides farinae and D. pteronyssinus (Acari: Pyroglyphidae): Direct Sequencing, Characterization and Polymorphism
Source: PLoS One. 2014 Dec 10;9(12):e114636. doi: 10.1371/journal.pone.0114636 (PMC4262422; doi:10.1371/journal.pone.0114636)

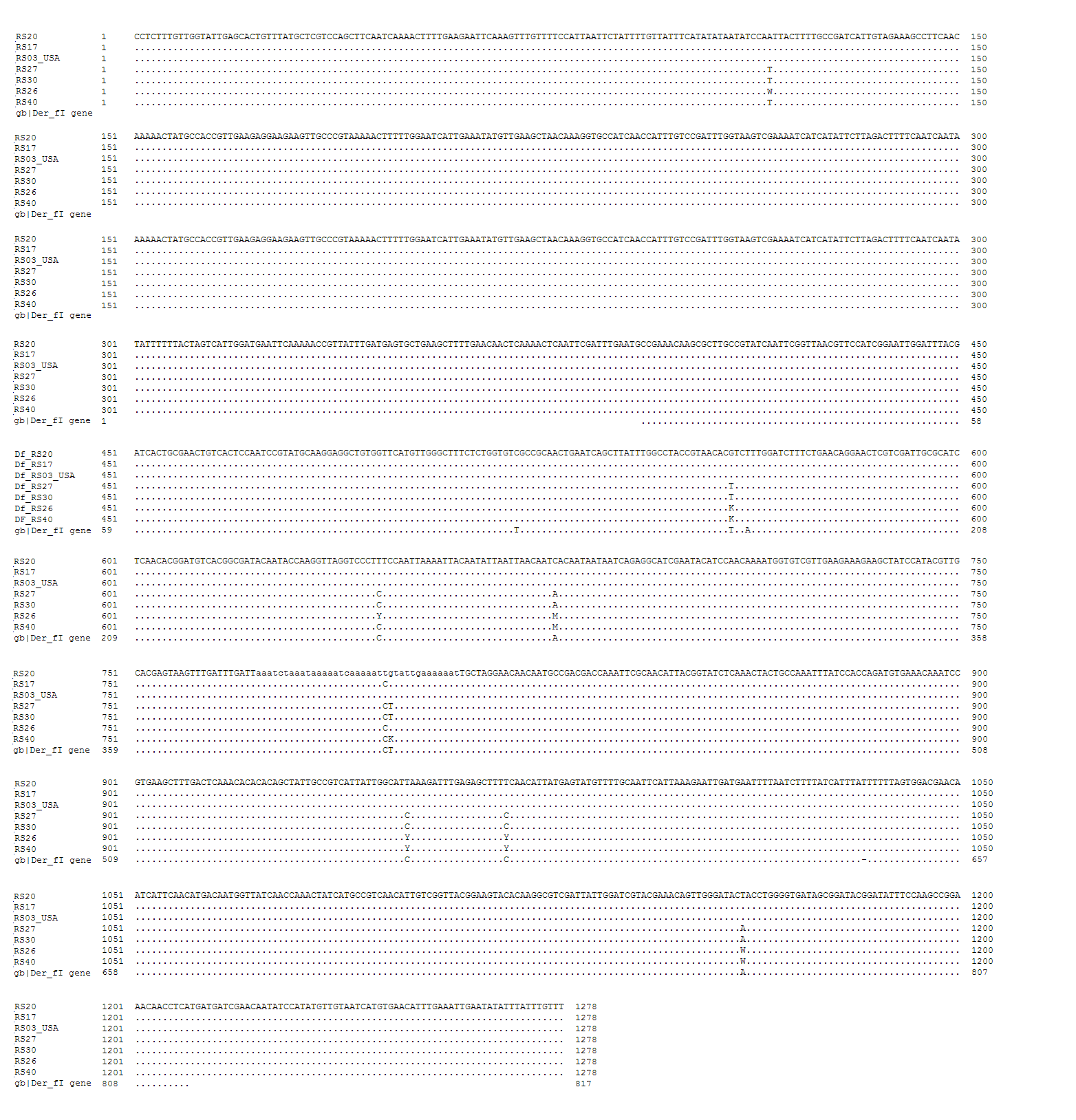

Supplement: S1 Figure — Selected sequences of the Der f 1 gene showing to two distinct haplotypes: Haplotype1-rows 1, 2 and 3 (RS20, RS17, RS03_USA); haplotype2-rows 4 and 5(Df_RS27 and Df_RS30); heterozygous - rows 6 and 7 (RS26 and RS40); and gb|Der-f1 gene (GenBank Accession number X65196). (TIF) [file pone.0114636.s001.tif]

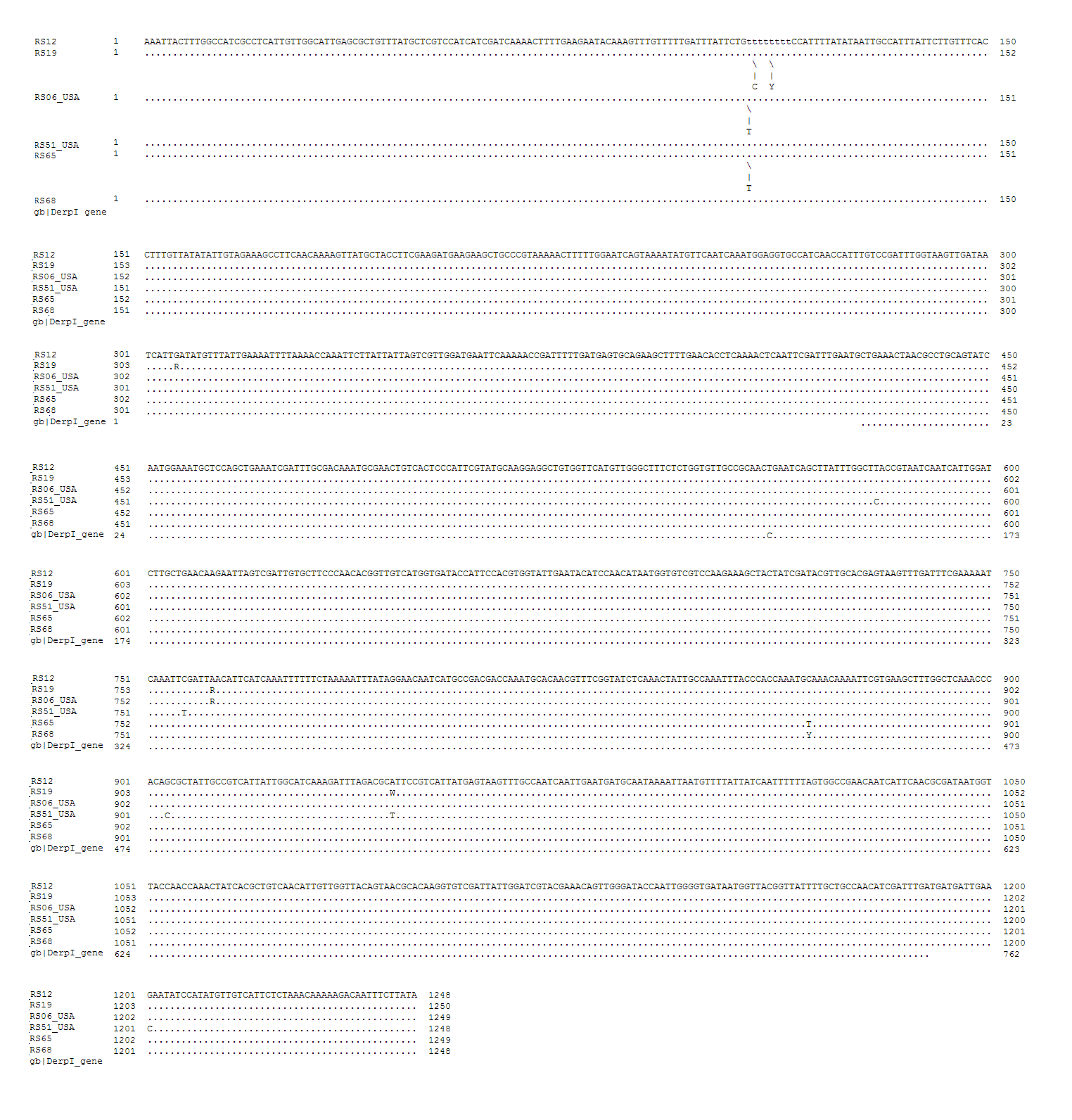

Supplement: S2 Figure — Selected Der p 1 gene sequences aligned in NCBI blastn. gb|Der-p1gene (GenBank accession number X65197.1). (TIF) [file pone.0114636.s002.tif]
